# Social and Metabolic Determinants of Prevalent Hypertension in Men and Women: A Cluster Analysis from a Population-Based Study

**DOI:** 10.3390/ijerph20031736

**Published:** 2023-01-18

**Authors:** Cornelia Bala, Adriana Rusu, Oana Florentina Gheorghe-Fronea, Theodora Benedek, Calin Pop, Aura Elena Vijiiac, Diana Stanciulescu, Dan Darabantiu, Gabriela Roman, Maria Dorobantu

**Affiliations:** 1 Department of Diabetes and Nutrition, “Iuliu Hatieganu” University of Medicine and Pharmacy, 400006 Cluj-Napoca, Romania; 2 Faculty of Medicine, University of Medicine and Pharmacy “Carol Davila”, 050474 Bucharest, Romania; 3 Cardiology Department, Clinical Emergency Hospital Bucharest, 014461 Bucharest, Romania; 4 Cardiology Department, County Clinical Emergency Hospital, Faculty of Medicine, “Gheorghe Emil Palade” University of Medicine, Pharmacy, Science, and Technology, 540142 Targu Mures, Romania; 5 Emergency Clinical County Hospital Baia Mare, Faculty of Medicine, “Vasile Goldiș” West University, 430130 Baia Mare, Romania; 6 Department of Cardiology, Arad Emergency Clinical County Hospital, Faculty of Medicine, “Vasile Goldiș” West University, 310025 Arad, Romania; 7 Romanian Academy, 010071 Bucharest, Romania

**Keywords:** hypertension, primary prevention, social determinants

## Abstract

Essential hypertension (HTN) has a complex spectrum of pathophysiological determinants and current guidelines provide limited information on high-risk groups that should be targeted for its primary prevention. The objective of our research was to identify clusters of social and metabolic factors associated with prevalent HTN in men and women from a population-based survey in Romania. Of the 1477 participants in the main study, 798 with complete data were analyzed here. Using two-step cluster analysis, one high-risk cluster in women and two high and intermediate risk for prevalent HTN in men were identified. Older age, rural area, lower education, and higher burden of metabolic factors characterized clusters with higher risk, while intermediate risk in men was characterized by a more metabolically healthy phenotype in younger individuals. In logistic regression, men in Cluster 1 vs. those in Cluster 3 had an odds ratio (OR) of 9.6 (95%CI: 4.6; 20.0), *p* < 0.001 for prevalent HTN, while OR for Cluster 2 vs. Cluster 3 was 3.2 (95%CI: 1.4; 7.4), *p* = 0.005. In women, the OR for HTN was 10.2 (95%CI: 5.7; 18.5) if assigned to Cluster 2 vs. Cluster 1, *p* < 0.001. These results pointed out the subgroups and communities that the primary prevention of HTN should be prioritized in.

## 1. Introduction

High blood pressure (BP), which includes hypertension (HTN), pre-hypertension, and other hazardous raises in BP, is responsible for over 8 million deaths globally from stroke, ischemic heart disease, other vascular diseases, and renal disease [1,2]. HTN is included among the four key metabolic changes that increase the risk of non-communicable diseases (NCDs) together with overweight/obesity, hyperglycemia, and hyperlipidemia [3]. Additionally, HTN is considered to be the most important modifiable determinant of atrial fibrillation and of its ischemic and hemorrhagic complications [4]. Moreover, HTN is demonstrated to be the leading metabolic risk factor with 19% attributed global deaths [3]. A target to lower the prevalence of raised blood pressure by 25% by 2025 compared with its 2010 level was proposed by the World Health Organization (WHO) to reduce premature mortality from NCDs by one third [5].

Recently reported data from a pooled analysis of 1201 population-representative studies with 104 million participants performed between 1990 and 2019 on people aged 30–79 years show disappointing results of the fight against HTN in terms of prevention, early detection, and treat-to-target measures. Although the global age-standardized prevalence of HTN remained stable between 1990 and 2019, the number of people with HTN doubled from 1990 to 2019 in both men and women as an effect of a decrease in HTN prevalence in high-income countries and an increase in some low- and middle-income countries. In 2019, only 59% of women and 49% of men with HTN reported a previous diagnosis of HTN. Similarly, only 47% of women and 38% of men were treated, while the control rates remain also low in 2019—23% for women and 18% for men, with large variations between regions and countries and despite improvements in most countries since 1990 [6]. These results point out that, in order to tackle HTN, a constellation of factors appertaining to national, community, and individual levels should be identified and addressed for the better prevention and treatment of HTN.

It is well-accepted and demonstrated that most of the cases of essential HTN occur in an environment of excessive central/visceral adipose tissue and insulin resistance that characterizes the metabolic syndrome, where high BP is associated with higher-than-normal levels of blood glucose and triglycerides and lower levels of high-density lipoprotein (HDL)-cholesterol [7]. More recently, the role of various social and economic factors in the relationship with HTN prevalence, awareness, treatment, and control are discussed as social determinants of HTN and as potential targets to improve primary and secondary prevention [8].

The identification of persons at high risk of HTN is a useful approach allowing targeted preventive and screening strategies. Various methods and models have been proposed for the identification of persons at a high risk of HTN. Among these the most widely tested were regression-based multifactorial risk models using as predictors the known risk factors of age, body mass index, previous diagnosis of diabetes, blood pressure, or genetic markers [9,10]. Although these models showed acceptable or good discriminative ability, and are assessable in clinical practice [9,10], they are applicable to individual persons and not to large groups. Thus, a method to identify large populational groups at risk of developing HTN using simple clinical and sociodemographic variables is needed and would allow a population-based targeted approach for primary preventative measures. Cluster analysis has been recently proposed as a new approach to characterize disease phenotypes and to identify subgroups of persons with similar characteristics at high risk of developing a disease or a complication of a disease [11]. The advantage of this technique is its ability to deal with large datasets, as seen in epidemiological studies, without requiring an investigator’s input on specific cut-off values for certain variables as required for risk model development [12].

Thus, the objective of our research is to identify clusters of social and metabolic factors associated with prevalent HTN in men and women using data from subjects included in a population-based survey from Romania, an Eastern European country with a high prevalence of HTN and cardiovascular diseases that has recently transitioned from being a middle-income to a high-income country.

## 2. Materials and Methods

The Study for the Evaluation of Prevalence of Hypertension and Cardiovascular Risk in Romania (SEPHAR) project includes 4 epidemiological cross-sectional studies with similar methodologies. Adults from 18 to 80 years of age randomly selected from the database of the Romanian population general direction of data records were invited to participate in these studies. As previously described [13], to ensure representability for the Romanian population, the recruitment was stratified by country regions, place of residence (rural and urban), gender, and age groups according to the data from the latest national census. Based on their response to the study invitation letter, the participants were enrolled and scheduled to undergo the medical evaluation in 1 of the 10 study centers across Romania. The only exclusion criterion was the refusal to participate. Here, we present an analysis of the fourth one performed between May and July 2020.

A full description of the methodology used in the SEPHAR IV survey was previously published [14,15]. Briefly, each participant in the SEPHAR IV study was evaluated through 2 study visits performed with 4-day intervals. During the first visit, data were collected on age, place of residence, gender, medical insurance status, education, personal and family medical history, anthropometric data (including weight, height, and waist circumference), BP (3 measurements performed at 1 min interval), cognitive status (Montreal Cognitive Assessment test), excessive daytime sleepiness (Epworth Sleepiness Scale), depression symptoms, and medication adherence (Morinsky scale). The ankle brachial index, arterial stiffness, and carotid B-mode Doppler were also performed as part of this visit. The second visit consisted in a measurement of BP, 12-leads ECG, standard transthoracic echocardiography, and collection of blood samples (including fasting glycemia, glycated hemoglobin A1c (HbA1c), total, LDL and HDL-cholesterol, and triglycerides).

During each visit, blood pressure was measured using an automated device certified by the Association for the Advancement of Medical Instrumentation, ESH, and the British Society of Hypertension (OMRON M6), according to the ESH and International Society of Hypertension recommendations [16]. For the analysis presented here, we used the mean of the second and third BP measurement during the first study visit as the BP value. Hypertension was defined as SBP ≥ 140 mmHg and/or a DBP ≥ 90 mmHg (the arithmetic mean of the second and third BP measurement of each study visit) or a previous diagnosis of hypertension.

The glycemic status was evaluated based on the fasting glycemia and HbA1c during the study visit and the self-reported medical history. Diabetes was defined as (1) a glycemia ≥ 126 mg/dl and an HbA1c ≥ 6.5% or (2) previous diagnosis of diabetes mellitus. Normoglycemia was defined as a glycemia < 110 mg/dl and an HbA1c < 5.7% and intermediate hyperglycemia as all cases not fulfilling the criteria for diabetes and normoglycemia. Abdominal obesity was defined as waist ≥ 80 cm in women and 94 cm in men.

All the participants provided a written informed consent before any study-related procedures. The SEPHAR IV study was approved by the Romanian National Ethics Committee (approval response no. 5S/4 from 24 September 2019).

### Statistical Analysis

The statistical analysis was performed using IBM SPSS Statistics version 26 (Armonk, NY, USA: IBM Corp). The variables were summarized using descriptive statistics: number and proportion for qualitative variables and median and quartiles 1 and 3 (Q1–Q3) for continuous variables. The independent-samples Mann–Whitney U and chi square tests were used for the variables between men and women.

To identify the participant subgroups with similar characteristics associated with a higher risk of HTN, we computed clusters separately for men and women based on sociodemographic, anthropometric, and metabolic factors: age, education, marital status, place of residence, health insurance status, abdominal obesity, overweight, glycemic status, smoking status, total cholesterol, LDL cholesterol, HDL cholesterol, triglycerides, and systolic and diastolic blood pressure as previously described [17]. The values of continuous variables were centered to a mean value of 0 and a standard deviation of 1. A two-step cluster analysis with log-likelihood as a distance measure and Schwarz’s Bayesian criterion (BIC) was used. This type of cluster analysis uses at first step a distance measure to separate groups, while during a second step a probabilistic method is used for hierarchical clustering for the estimation of the optimal number of clusters [18,19]. Due to the use of statistical measures of fit for determining the number of clusters and handling both continuous and categorical data, this method is considered as one of the most reliable clustering techniques for the number of clusters chosen, classification of cases, and reproducibility of findings [18,19,20].

For a detailed description of the identified clusters, a quantitative comparison of the socio-demographic, anthropometric, and metabolic characteristics between clusters was performed separately in men and women using Kruskal–Wallis nonparametric test.

Based on the similarity of sociodemographic, anthropometric, and metabolic factors used in the cluster analysis, each case was assigned to a cluster and the cluster was used as a predictor for the presence of hypertension using the logistic regression. The outliers were excluded from this analysis. For all the analyses, a *p*-value < 0.05 was considered statistically significant.

## 3. Results

Of the 1477 participants to the SEPHAR IV study, 679 were excluded due to missing age, gender, marital status, residential status, smoking, waist, height, weight, or complete data on glucose and/or lipid profile. Thus, 798 participants with complete date were analyzed. The women represented 57.9% of the study sample, with most participants living in urban areas and having secondary school or university as the highest education level. Additionally, over half of the sample were overweight or had obesity and abdominal obesity. The prevalence of diabetes was 10.4%, that of intermediate hyperglycemia 31.5%, and the prevalence of arterial hypertension was 42.4%. The men had significantly higher levels of SBP and DBP than women, although for the prevalence of arterial hypertension no difference between genders was observed. Additionally, the men were more frequently unmarried, had significantly higher levels of triglycerides and BMI, were more frequently overweight or obese, and were former or current smokers. No other differences between genders were observed as depicted in Table 1.

**Figure 1:**
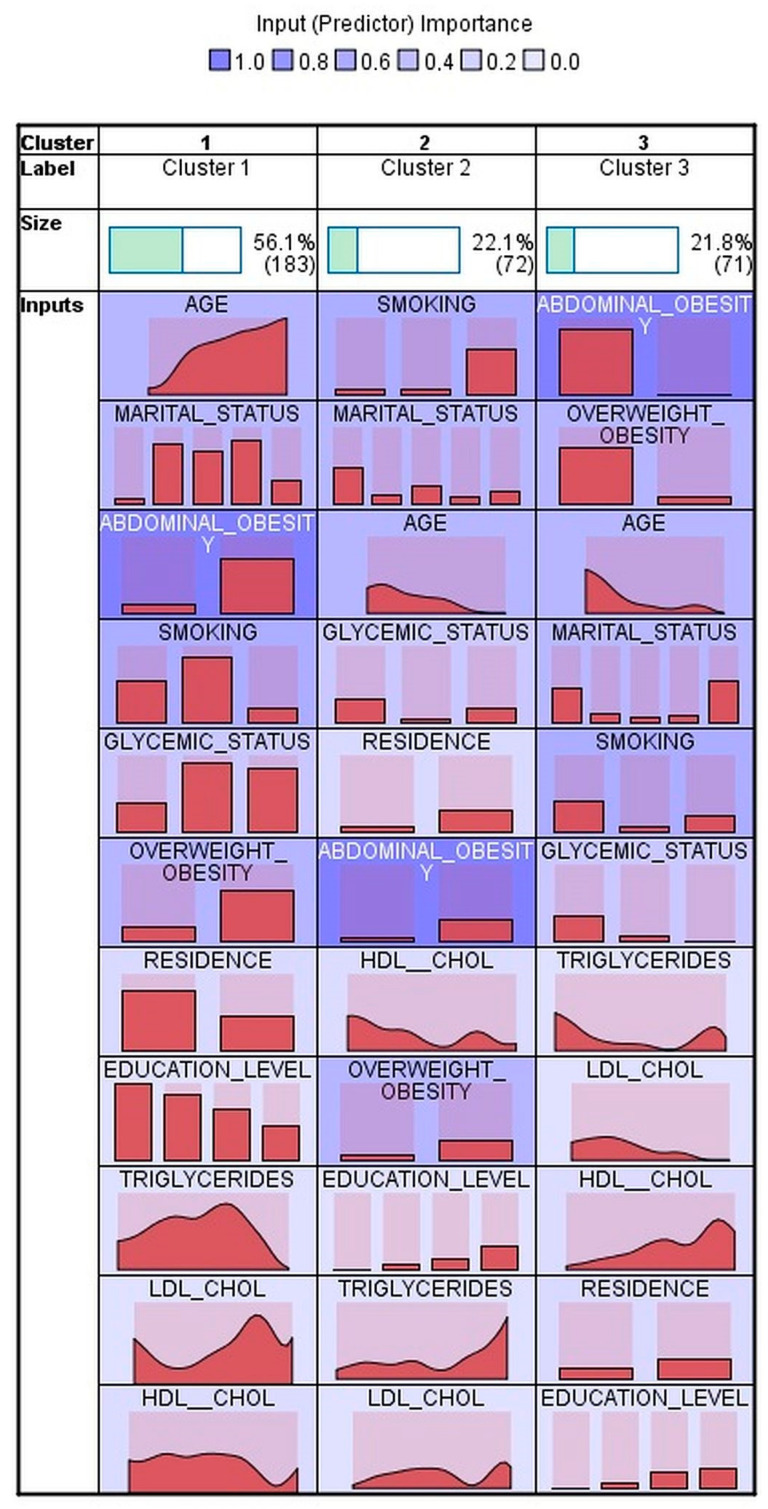
Clusters in men. Input variables are sorted within cluster importance. Cells show relative distribution of input values.

**Figure 2:**
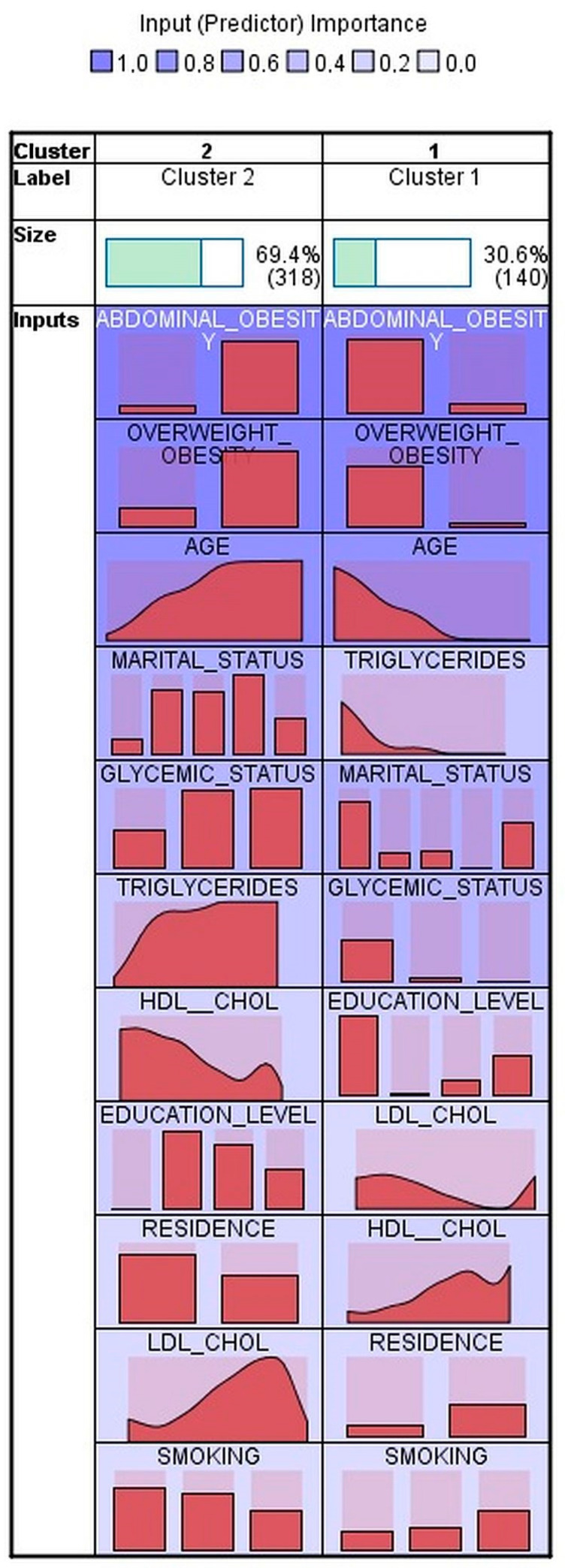
Clusters in women. Input variables are sorted within cluster importance. Cells show relative distribution of input values.

Using two-step cluster analysis, three clusters in men (Figure 1) and two clusters in women (Figure 2) were identified of fair quality both in men and in women. The health insurance status was not entered into the cluster analysis, as the vast majority of the participants reported having health insurance coverage. The socio-demographic, anthropometric and metabolic characteristics, according to cluster allocation, are presented in Table 2 for men and in Table 3 for women. The number of outliers was small in both men—10 (3%) and women—4 (0.9%); those participants were excluded from comparison between clusters analyses.

In men, the frequency of arterial HTN was 61.2, 34.7, and 14.1% in Clusters 1, 2, and 3, respectively, and, in women, the frequency of HTN was 14.0 and 53.2% in Clusters 1 and 2.

In men, the lowest prevalence of HTN was found in Cluster 3—71 subjects (21.8%)—in whom abdominal obesity was virtually absent, overweight/obesity less frequent, of young age, more frequently unmarried, never smokers, with normoglycemia in most cases, lower levels of triglycerides and LDL-cholesterol, higher levels of HDL-cholesterol, more frequently having urban residence, and with higher education levels (secondary school or university). The highest prevalence of HTN was found in individuals from Cluster 1, which included 183 (56.1%) men and who were older, more frequently married, almost all have abdominal obesity, more frequently ex-smokers, with intermediate hyperglycemia or diabetes in more than 50% of cases, with overweight/obesity, and residing in rural areas. The men in Cluster 1 also had higher levels of triglycerides and LDL-cholesterol and lower HDL-cholesterol. The men allocated to Cluster 2 also had a significantly higher prevalence of HTN compared to Cluster 3. They had a younger age, close to the median age in Cluster 3, but with high prevalence of abdominal obesity and overweight/obesity while the other metabolic characteristics were intermediate between those seen in Cluster 3 and 1, except normoglycemia, which was seen in a quite large number (86.1%) of cases. This cluster included the highest percentage of smokers and of urban residents (Table 2).

In women, two clusters were generated—Cluster 1 with 140 (30.6%) subjects and Cluster 2 with 318 (69.4%) subjects. Cluster 2 included women with a high prevalence of HTN who were older, with high percentages of abdominal obesity and overweight/obesity, more frequently married, never smokers, less education, almost half from rural areas, with higher levels of triglycerides and LDL-cholesterol, lower levels of HDL-cholesterol, and with high percentage of intermediate hyperglycemia and diabetes as opposed to those in Cluster 1 (Table 3).

In the logistic regression, the men in Cluster 1 vs. those in Cluster 3 had an odds ratio (OR) of 9.6 (95%CI: 4.6; 20.0), *p* < 0.001 to have prevalent HTN, while OR for Cluster 2 vs. Cluster 3 was 3.2 (95%CI: 1.4; 7.4), *p* = 0.005. In women, the OR of having HTN was 10.2 (95%CI: 5.7; 18.5) if assigned to Cluster 2 vs. Cluster 1, *p* < 0.001.

## 4. Discussion

In this post hoc analysis from a population-based study performed in Romania, we report that individuals from a general population cohort can be grouped in three clusters of men and two clusters of women according to their socio-demographic and metabolic characteristics and that some of these clusters are strongly correlated with prevalent HTN.

The essential HTN has a complex spectrum of pathophysiological determinants—genetic and epigenetic factors, environmental factors, and social determinants with multiple interactions between these three categories of risk factors [21].

Such a complex network of risk factors would impose complex strategies for HTN prevention and control and the most effective strategy to reduce HTN-related complication would be to prevent the occurrence of HTN (primary prevention) using a combination of general population interventions and targeted interventions in high-risk groups or communities [21,22,23]. In Romania, an Eastern European country recently transitioned from being a middle- to a high-income country; the prevalence of HTN between 2005–2016 varied between 40 and 45% with no trend toward a decrease, even though HTN awareness increased throughout this period [24]. Therefore, more intensive and better-informed prevention programs are deemed to be urgently needed.

The guidelines on the prevention and treatment of HTN designate high-risk individuals as those of certain ethnic origins (e.g., African Americans), those who are overweight, those who consume excessive amounts of dietary sodium, have a high intake of alcohol, or are physically inactive [16,22,25,26], but for practical prevention purposes these recommendations may be too general to inform clinicians on the characteristics of communities or groups with a higher risk of developing HTN and where primary prevention programs may be most beneficial.

Cluster analysis is a statistical method that allocates subjects into groups (clusters) based on a pre-selected set of data/characteristics such that the subjects in each cluster are more similar to each other than to those in other clusters. It has the advantage of not requiring pre-specified thresholds or cut-off values and it is considered a suitable mean to deal with large datasets of patients/subjects that have numerous inherent characteristics that in turn can influence health outcomes [12].

In HTN research, this method was mainly used to identify phenotypes of hypertensive subjects that have different risks for developing cardiovascular complications [17,27,28]. An exploratory analysis of the Systolic Blood Pressure Intervention Trial (SPRINT) reported that subjects included in the trial can be grouped in four clusters where the fourth one did not differ significantly in BMI and eGFR but had a higher Framingham risk score than other clusters. This cluster had the highest risk for a composite of non-fatal myocardial infarction (MI), acute coronary syndrome not resulting in an MI, non-fatal stroke, non-fatal acute decompensated HF, and death from cardiovascular causes and it was also the only cluster where intensive antihypertensive treatment was shown to be beneficial [28].

In the analyses reported here, we used the advantages of cluster analysis on a dataset of demographic, social, and metabolic characteristics of individuals representative for a general population included in the SEPHAR IV study with the aim to identify subgroups at a higher risk for prevalent HTN. We chose to perform a separate analysis in men and women taking into consideration the recent findings that blood pressure should be considered a sexually dimorphic trait due to several distinctive features between men and women (e.g., in women there is a much sharper incline in blood pressure from the third decade of life and they are at higher risk of developing adverse cardiovascular events at lower blood pressure thresholds) [29].

In women, two distinct clusters were generated based on the input variables of age, education level, marital status, smoking, presence of overweight/obesity, of abdominal obesity, dysglycemia (either diabetes or intermediate hyperglycemia), and levels of triglycerides, HDL-cholesterol, and LDL-cholesterol. The women in Cluster 2, representing 69.4% of the female population, are older (median age 59.1 years), are overweight/have obesity in over 87% of cases and abdominal obesity in over 97% of cases, and display metabolic features that are characteristic for the metabolic syndrome. They are more often from rural areas, have a lower level of education, and have never smoked in 67% of cases. The women in Cluster 1 have, in general, opposite characteristics, being much younger and with only 10.7% and 26.4% being overweight/having obesity and abdominal obesity, respectively. The OR for prevalent HTN is 10.2 in Cluster 2 vs. Cluster 1.

The male subjects were grouped in three clusters based on the same input variables. The men in Cluster 1, representing 56.1% and with a median age of 57.4 years, have similar metabolic and social characteristics as the women in Cluster 2 and an OR for prevalent HTN of 9.6 compared with Cluster 3 (21.8% of male subjects, median age 30.5%). In Cluster 3, abdominal obesity is virtually absent and overweight/obesity affects only 33.8% of the subjects allocated to this cluster.

Thus, two similar clusters were identified in men and women, both associated with a high risk of prevalent HTN and both sharing similar anthropometric characteristics and social determinants—older age, high frequency of abdominal obesity and overweight/obesity, residence in the rural area, lower educational level, and a higher frequency of features that are characteristic for the metabolic syndrome (low HDL cholesterol, high triglycerides, and higher glycemia).

Overweight/obesity and central adiposity are well-recognized factors for the development of HTN. In a meta-analysis of more than 2.3 million participants from 57 prospective studies, the relative risk (RR) to develop HTN was 1.49 for a five-unit increment in BMI, 1.27 for a 10 cm increment in waist circumference, and 1.16 for a weight gain equal to a one-unit increment in BMI [25]. This relationship was also previously demonstrated in our population using data from the SEPHAR III study, where both the BMI and waist circumference were significant predictors of prevalent HTN [26]. Similar to numerous other studies, the individuals with HTN in SEPHAR III also had other factors included in the metabolic syndrome—higher serum triglycerides and blood glucose and lower levels of HDL-cholesterol [26]. Other environmental factors related to the risk of HTN are unhealthy diet and physical inactivity, which in most part act through promoting excessive adiposity, high sodium intake, low potassium intake, and alcohol consumption.

Social determinants are more recently discussed as risk factors for HTN and most data on their role in mediating incidental HTN come from high-income countries. In a conceptual framework, the social determinants of hypertension were categorized in structural—including socio-economic and political context, gender, ethnicity, and socio-economic status (education, income, and occupation)—and intermediary factors—material circumstances, behavioral, psychosocial, and biological factors [8]. The association between a lower educational level and a high risk of HTN has been previously proven in high-income countries [30,31] and can be explained by a lower awareness and poorer treatment and control of HTN among those with lower educational attainment [32,33]. Residence in rural areas has been reported as being associated with a higher prevalence of HTN than urban areas only in developed regions [34,35]. The potential explanations for these observations are the economic development and urbanization of rural areas as well as the associated lifestyle changes (unhealthy diet and physical inactivity) that are not backed-up by proper health literacy and the limited access to healthcare [34,35]. Additionally, for Romania, it has been shown that there is a higher prevalence of overweight people and obesity in the rural areas [36], a higher alcohol consumption compared to the urban ones, and an unhealthy diet characterized by a high intake of meat and dietary fats and a lower intake of fresh vegetables and fruits (thus a low potassium intake) [37,38].

Interestingly, 22.1% of men are allocated to a third cluster (Cluster 2) where the median age is closer to that in Cluster 3—36.4 years but with high percentages affected by abdominal obesity (94.4%) and overweight/obesity (93.1%) similar to those seen in Cluster 1. Despite excessive central and total adiposity, the men in Cluster 2 have normoglycemia in 86.1% of cases and intermediate levels of triglycerides, LDL-, and HDL-cholesterol compared with Clusters 1 and 3. Nevertheless, the OR for prevalent HTN is significantly higher (3.2 [95%CI: 1.4; 7.4], *p* = 0.005) compared to Cluster 3. We can hypothesize that the men in Cluster 3 are those progressing from a metabolically healthy obesity (MHU) to a metabolically unhealthy obesity (MUO). It is now well-recognized that MHU is only a transient phase in the trajectory of a patient with obesity and that continuous exposure to excessive adiposity over time will lead to progression to MUO where the burden of metabolic factors and of cardiovascular risk is increased [39,40].

In both men and women, marital status according to cluster allocation and further on with the risk of HTN yielded confusing results as the percentage of subjects who are married is notably higher in clusters at risk for HTN. Marital status was chosen as a substitute for social isolation/loneliness, the latter being discussed as one of the social determinants of hypertension. Previous studies used the Berkman social network index (SNI), which assesses the degree of social integration by calculating the number of social ties across five domains: marital status, contact with parents, contact with child/children, contact with neighbors, and volunteer activities. Only subjects with four or five ties had a lower risk of HTN [41]. Therefore, marital status alone may not be sufficient to characterize the absence or presence of social isolation. Additionally, in older groups, at least in traditional societies, the percentage of married individuals tends to be higher.

As mentioned before, cluster analysis was mainly used in hypertensive populations in relation with HTN-related outcomes. A somehow similar study to our research was performed in women with pre-eclampsia who were evaluated for the risk of developing persistent post-partum hypertension (PHTN) [42]. In this study, three clusters were associated with high-, medium-, and low-risk PHTN and women in the high-risk cluster were characterized as being older, with higher BMI and mean arterial pressure values, having used antihypertensive drugs before delivery, with abnormal pregnancy features, are multiparous, with longer intervals between births, and with worse laboratory results.

Taken individually, the socio-demographic and metabolic characteristics displayed by men or women allocated to higher-risk clusters in our study were already known as being associated with the risk of HTN. We consider that the strength of our analysis is that, using cluster analysis, we were able to group the individuals from a general population according to multiple features into clusters associated with different levels of risk for prevalent HTN that can further inform clinicians to better target individuals and sub-groups at risk. We also acknowledge that the cross-sectional nature of our study as well as the limited number of social characteristics included in the analysis represent a limitation in establishing definite conclusions, but our results point out that communities from rural areas and/or with lower level of education are more prone to have more obesity and more deleterious glycemic and lipid factors that are associated with increased risk of prevalent and probably incident HTN and that these communities should be prioritized for primary prevention actions. Additionally, men in their fourth decade of age who have abdominal obesity and overweight/obesity should also be considered for the prevention of HTN even in the absence of established features of the metabolic syndrome.

## 5. Conclusions

In this analysis of a cohort of the general population, we identified distinct subgroups of men and women with higher and lower risks for prevalent HTN based on social and metabolic determinants. Men and women from rural areas and/or lower levels of education had higher levels of adiposity and glucose and lipid disturbances and men in their fourth decade with a phenotype corresponding to metabolically healthy obesity are the groups with a significantly higher risk of prevalent HTN. The results of this analysis have public health and clinical significance. They show the possibility to identify subgroups at a high risk of HTN, thus allowing for a targeted approach for primary prevention measures before the development of arterial HTN, screening, and interventions. The interventions targeting cluster membership may allow for a more efficient healthcare and health promotion allocation of the resources. For clinicians, the recognition of different subgroups with a high risk of HTN based on comorbidities and social determinants may help in choosing appropriate management approaches for each case to prevent, diagnose, and control HTN and associated conditions. Although our results are based on data from a specific European population that has undergone a recent transition from being a middle- to high-income country, our results should be confirmed in other European populations.

## Figures and Tables

**Table 1:** Sociodemographic, anthropometric, and metabolic characteristics of participants included in the analysis.

	Total N = 798	Men N = 336	Women N = 462	*p*-Value
Age, years	52.1 (37.5; 65.3)	51.2 (33.8; 65.7)	52.5 (39.0; 64.8)	0.179
Urban residence, n (%)	501 (62.8%)	222 (66.1%)	279 (60.4%)	0.103
Education, n (%) No studies Primary school Secondary school University	2 (0.3%) 59 (7.4%) 388 (48.6%) 349 (43.7%)	1 (0.3%) 19 (5.7%) 153 (45.5%) 163 (48.5%)	1 (0.2%) 40 (8.7%) 235 (50.9%) 186 (40.3%)	0.086
Marital status, n (%) Unmarried Married Factual union Divorced Widow	182 (22.8%) 482 (60.4%) 49 (6.1%) 61 (7.6%) 24 (3.0%)	90 (28.8%) 204 (60.7%) 16 (4.8%) 12 (3.6%) 14 (4.2%)	92 (19.9%) 278 (60.2%) 33 (7.1%) 49 (10.6%) 10 (2.2%)	<0.001
Health insurance, n (%)	758 (95.0%)	326 (97.0%)	432 (93.5%)	0.032
SBP, mmHg	127.0 (114.0; 139.0)	133.0 (123.0; 142.0)	122.0 (110.0; 134.0)	<0.001
DBP, mmHg	81.0 (74.0; 89.0)	83.0 (76.0; 91.0)	79.0 (72.0; 88.0)	<0.001
Hypertension, n (%)	338 (42.4%)	154 (45.8%)	184 (39.8%)	0.095
Waist, cm	97.9 (85.0; 108.0)	103.0 (92.0; 112.0)	93.0 (80.0; 103.0)	<0.001
Abdominal obesity, n (%)	596 (74.7%)	248 (73.8%)	348 (75.3%)	0.680
BMI, kg/m^2^	28.1 (24.2; 31.9)	28.4 (25.9; 31.4)	27.5 (23.3; 32.0)	0.008
Overweight and obesity, n (%)	562 (70.4%)	267 (79.5%)	295 (63.9%)	<0.001
Cholesterol, mg/dl	198.5 (172.0; 230.0)	198.0 (174.0; 223.5)	200.0 (171.0; 233.0)	0.432
HDL cholesterol, mg/dl	52.0 (44.0; 63.0)	48.0 (40.0; 55.0)	56.0 (47.0; 66.0)	<0.001
LDL cholesterol, mg/dl	128.5 (100.0; 160.0)	130.0 (104.5; 160.0)	127.0 (99.0; 159.0)	0.385
Triglycerides, mg/dl	99.5 (68.0; 149.0)	117.0 (82.5; 175.5)	90.0 (62.0; 129.0)	<0.001
Fasting glycemia, mg/dl	95.0 (88.0; 104.5)	95.5 (89.0; 106.0)	95.0 (87.0; 104.0)	0.213
HbA1c, %	5.5 (5.2; 5.8)	5.4 (5.2; 5.8)	5.5 (5.2; 5.9)	0.017
Glycemic status, n (%) Normoglycemia Intermediate hyperglycemia Diabetes	464 (58.1%) 251 (31.5%) 83 (10.4%)	205 (61.0%) 96 (28.6%) 35 (10.4%)	259 (56.1%) 155 (33.5%) 48 (10.4%)	0.308
Smoking status, n (%) Never smoker Former smoker Current smoker	399 (50.0%) 185 (23.2%) 214 (26.8%)	120 (35.7%) 120 (35.7%) 96 (28.6%)	279 (60.4%) 65 (14.1%) 118 (25.5%)	<0.001

Data in table are presented as median (Q1–Q3) for continuous variables and as number (%) for categorical variables. N/n (%) = number (percentage); SBP = systolic blood pressure; DBP = diastolic blood pressure; BMI = body mass index; and HbA1c = glycated hemoglobin.

**Table 2:** Distribution of sociodemographic, anthropometric, and metabolic characteristics of participants according to clusters identified in men.

	Men N = 336			
	Cluster 1 N = 183	Cluster 2 N = 72	Cluster 3 N = 71	*p*-Value
Age, years	57.4 (47.5; 69.2)	36.4 (29.0; 59.1)	30.5 (25.5; 45.5)	<0.001
Residence, n (%) Rural Urban	83 (45.4%) 100 (54.6%)	8 (11.1%) 64 (88.9%)	16 (22.5%) 55 (77.5%)	<0.001
Education, n (%) No studies Primary school Secondary school University	1 (0.5%) 11 (6.0%) 99 (54.1%) 72 (39.3%)	0 (0.0%) 1 (1.4%) 21 (29.2%) 50 (69.4%)	0 (0.0%) 1 (1.4%) 30 (42.3%) 40 (56.3%)	<0.001
Marital status, n (%) Unmarried Married Factual union Divorced Widow	6 (3.3%) 155 (84.7%) 9 (4.9%) 9 (4.9%) 4 (2.2%)	43 (59.7%) 23 (31.9%) 3 (4.2%) 1 (1.4%) 2 (2.8%.)	41 (57.7%) 21 (29.6%) 1 (1.4%) 1 (1.4%) 7 (9.9%)	<0.001
Abdominal obesity, n (%)	174 (95.1%)	68 (94.4%)	1 (1.4%)	<0.001
Overweight and obesity, n (%)	171 (93.4%)	67 (93.1%)	24 (33.8%)	<0.001
HDL cholesterol, mg/dL	47.0 (40.0; 54.0)	45.5 (37.0; 50.0)	53.0 (46.0; 64.0)	<0.001
LDL cholesterol, mg/dL	138.0 (107.0; 172.0)	131.0 (112.25; 151.75)	113.0 (91.0; 139.0)	0.001
Triglycerides, mg/dL	134.0 (90.0; 184.0)	120.5 (83.5; 183.5)	84.0 (58.0; 121.0)	<0.001
Glycemic status, n (%) Normoglycemia Intermediate hyperglycemia Diabetes	76 (41,5%) 79 (43.2%) 28 (15.3%)	62 (86.1%) 4 (5.6%) 6 (8.3%)	66 (93.0%) 5 (7.0%) 0 (0%)	<0.001
Smoking status, n (%) Never smoker Former smoker Current smoker	63 (34.4%) 102 (55.7%) 18 (9.8%)	7 (9.7%) 9 (12.5%) 56 (77.8%)	45 (63.4%) 7 (9.9%) 19 (26.8%)	<0.001

Data in table are presented as median (Q1–Q3) for continuous variables and as number (%) for categorical variables. N/n (%) = number (percentage).

**Table 3:** Distribution of sociodemographic, anthropometric, and metabolic characteristics of participants according to clusters identified in women.

	Women N = 458		
	Cluster 1 N = 140	Cluster 2 N = 318	*p*-Value
Age, years	34.4 (29.9: 46.7)	59.1 (49.6; 69.3)	<0.001
Residence, n (%) Rural Urban	26 (18.6%) 114 (81.4%)	153(48.1%) 165 (51.9%)	<0.001
Education, n (%) No studies Primary school Secondary school University	1 (0.7%) 1 (0.7%) 44 (31.4%) 94 (67.1%)	0 (0.0%) 38 (11.9%) 188 (59.1%) 92 (28.9%)	<0.001
Marital status, n (%) Unmarried Married Factual union Divorced Widow	75 (53.6%) 53 (37.9%) 7 (5.0%) 0 (0.0%) 5 (3.6%)	16 (5.0%) 223 (70.1%) 26 (8.2%) 49 (15.4%) 4 (1.3%)	<0.001
Abdominal obesity, n (%)	37 (26.4%)	309 (97.2%)	<0.001
Overweight and obesity, n (%)	15 (10.7%)	278 (87.4%)	<0.001
HDL cholesterol, mg/dL	65.0 (52.3; 73.0)	53.0 (46.0; 62.0)	<0.001
LDL cholesterol, mg/dL	112.0 (88.3; 132.0)	134.0 (105.0; 169.3)	<0.001
Triglycerides, mg/dL	61.5 (50.3; 81.5)	104.0 (73.0; 149.8)	<0.001
Glycemic status, n (%) Normoglycemia Intermediate hyperglycemia Diabetes	134 (95.7%) 6 (4.3%) 0 (0.0%)	123 (38.7%) 147 (46.2%) 48 (15.1%)	<0.001
Smoking status, n (%) Never smoker Former smoker Current smoker	63 (45.0%) 19 (13.6%) 58 (41.4%)	214 (67.3%) 46 (14.5%) 58 (18.2%)	<0.001

Data in table are presented as median (Q1–Q3) for continuous variables and as number (%) for categorical variables. N/n (%) = number (percentage).

## Data Availability

Data can be requested from the corresponding author.
